# What Makes Children Defy Majorities? The Role of Dissenters in Chinese and Spanish Preschoolers’ Social Judgments

**DOI:** 10.3389/fpsyg.2016.01695

**Published:** 2016-10-27

**Authors:** Ileana Enesco, Carla Sebastián-Enesco, Silvia Guerrero, Siyu Quan, Sonia Garijo

**Affiliations:** ^1^Departamento de Psicología Evolutiva y de la Educación, Facultad de Psicología, Universidad Complutense de MadridMadrid, Spain; ^2^William James Center for Research, Instituto Superior de Psicologia Aplicada – Instituto UniversitárioLisboa, Portugal; ^3^Departamento de Psicología, Facultad de Educación, Universidad de Castilla-La ManchaToledo, Spain

**Keywords:** social development, moral judgment, cross-cultural psychology, testimony, majority, dissenter, consensus

## Abstract

When many people say the same thing, the individual is more likely to endorse this information than when just a single person says the same. Yet, the influence of consensus information may be modulated by many personal, contextual and cultural variables. Here, we study the sensitivity of Chinese (*N* = 68) and Spanish (*N* = 82) preschoolers to consensus in social decision making contexts. Children faced two different types of peer-interaction events, which involved (1) uncertain or ambiguous scenarios open to interpretation (social interpretation context), and (2) explicit scenarios depicting the exclusion of a peer (moral judgment context). Children first observed a video in which a group of teachers offered their opinion about the events, and then they were asked to evaluate the information provided. Participants were assigned to two conditions that differed in the type of consensus: Unanimous majority (*non-dissenter* condition) and non-unanimous majority (*dissenter* condition). In the *dissenter* condition, we presented the conflicting opinions of three teachers vs. one teacher. In the *non-dissenter* condition, we presented the unanimous opinion of three teachers. The general results indicated that children’s sensitivity to consensus varies depending both on the degree of ambiguity of the social events and the presence or not of a dissenter: (1) Children were much more likely to endorse the majority view when they were uncertain (social interpretation context), than when they already had a clear interpretation of the situation (moral judgment context); (2) The presence of a dissenter resulted in a significant decrease in children’s confidence in majority. Interestingly, in the moral judgment context, Chinese and Spanish children differed in their willingness to defy a majority whose opinion run against their own. While Spanish children maintained their own criteria regardless of the type of condition, Chinese children did so when an “allied” dissenter was present (dissenter condition) but not when confronting a unanimous majority (non-dissenter condition). Tentatively, we suggest that this difference might be related to culture-specific patterns regarding children’s deference toward adults.

## Introduction

Much of our knowledge is acquired from the testimony of others and not from our direct experience. Historical facts, scientific constructs, religious beliefs, social values and norms, for example, are types of human knowledge mainly formed from testimonial sources. But what makes us accept information provided by others? One of the variables is the degree of consensus, that is, how many people say the same thing or hold the same view. The influence of majority on adults’ decisions or behaviors has been extensively studied since Asch (1956) seminal work on conformity (Bond and Smith, 1996). Broadly speaking, the findings show that the influence of majority varies according not only to the degree of consensus (e.g., unanimous vs. partial majorities), but also to other variables, such as an individual’s prior knowledge (e.g., naïve vs. experienced individuals), the ambiguity of the task (e.g., a situation more or less open to interpretation), and various situational determinants (e.g., public or anonymous responses). Thus, the strength of the majority is greater when unanimous, the task is new or ambiguous, and the individual’s response is public (see Cialdini and Goldstein, 2004; Haun et al., 2013 for a review). Another source of variability in the way individuals deal with the testimony of others is attributed to culture. Some cross-cultural studies have reported that adults socialized in East Asian countries tend to rely on consensus more than adults socialized in Western post-industrial countries (Huang and Harris, 1973; Fiske et al., 1998; Kim and Markus, 1999).

Studies conducted in adults far outnumber those conducted in children. To date, research on children’s sensitivity to consensus has focused on limited fields of knowledge (vocabulary, objects functions, perceptual judgments, and physical laws) and has been based on two main approaches. Some researchers have studied the influence of consensus or numerical majorities on children’s endorsement of new information, such as the name of a new object (Corriveau et al., 2009; Chen et al., 2011; Cascado et al., 2014; Bernard et al., 2015; Guerrero et al., in press) or the function of unfamiliar objects (Birch et al., 2008). Within this line of research, a common experimental scenario is the conflicting-claims paradigm (Koenig et al., 2004) in which different informants express different views about a particular situation, and the child is asked to decide between the options. A number of other works have assessed children’s sensitivity to consensus when they have prior beliefs or knowledge about the issue and the assertion of a majority of informants contradicts these beliefs. This is based on the Asch’s paradigm, where the individual is faced with a majority that makes an obviously erroneous judgment about a very simple perceptual experience (e.g., judging the relative lengths of three lines, Corriveau and Harris, 2010; Hanayama and Mori, 2011; Haun and Tomasello, 2011) or about implausible functions of familiar objects (Seston Schillaci and Kelemen, 2014). Both types of approaches address whether children show any bias toward majorities, that is, whether they accept or reject the specific information provided by the informants.

In general, most of the findings reveal that, from about age 4, children tend to endorse majority testimony on new information, such as labels and functions for unfamiliar or ambiguous objects, whereas they are reluctant to side with a majority making a judgment that is obviously wrong. For example, in the study conducted by Corriveau et al. (2009), when deciding the label for a new object, children tended to select the one proposed by the majority in about 65% of the trials; by contrast, in studies based on Asch’s paradigm, less than 30% of the children’s choices sided with an “erroneous” majority (Corriveau and Harris, 2010; Hanayama and Mori, 2011; Haun and Tomasello, 2011) (for a critical review, see Hodges, 2014). All these findings suggest that children’s knowledge base modulates their sensitivity to the testimony of others, at least in the epistemic contexts studied in previous research. However, studies with children are still scarce and many questions remained unanswered. For the purposes of the current study, we outline three aspects that deserve further attention: the influence of consensus on children’s decisions about social matters, a domain particularly neglected in past research; the role of dissenters, that is, the presence of an informant who opposes the majority view; and the role of children’s cultural background. We briefly discuss these aspects.

The social domain is, for various reasons, an interesting context in the study of the testimony of others. First, social events are, by their very nature, subject to interpretation in the sense that help may be required from others to understand their meaning (for example, how to interpret an episode involving two actors, one crying, the other one just watching?). In many cases, the rules and motives underlying social interactions are opaque. Second, the acquisition of social norms and values takes place through interaction and negotiation with others, who sometimes hold different or even opposing views. And third, children of 4–5 years already have knowledge about a wide range of social matters: They are able to judge familiar social interactions according to intentions and not merely outcomes; they apply moral concepts (such as fairness in peer exchanges or empathy toward a victim) and non-moral concepts (e.g., group functioning, personal prerogatives) depending on the nature of the events (Nucci, 2001; Turiel, 2005). Thus, an interesting question here is how children assimilate the testimony of others when deciding about familiar and routine social interactions. To our knowledge, only a few studies have addressed preschoolers’ trust in consensus in contexts of moral decision, presenting adults as informants (Guerrero et al., in press, with Spanish participants) or peers as informants (Kim et al., 2016, with American participants). Although we cannot directly compare these studies due to the large methodological differences, their general findings revealed that preschoolers were prone to oppose to a ‘morally wrong’ consensus, though the rate of resistance to consensus was higher among the Spanish preschoolers compared to the Americans. However, in the Guerrero et al. (in press) study there was always a dissenter whose view coincided with that of the children. We do not know if children maintain the same position without the presence of an “allied” dissenter.

There is little research on the role of dissenters in the testimony of others. Authors have usually addressed the influence of consensus by presenting either a unanimous majority, as in the perceptual judgment paradigm (Corriveau and Harris, 2010; Haun and Tomasello, 2011), or a majority versus a dissenter, a standard condition for the novel object labeling paradigm (Corriveau et al., 2009). Among the few studies comparing unanimous and non-unanimous majorities, Morgan et al. (2015) presented US children aged 3–7 years with tasks of quantity discrimination, varying the degree of consensus among the informants. Most children trusted the information provided by unanimous majorities, but only the older children were also influenced by non-unanimous majorities. In the context of learning non-conventional functions for familiar objects, Cascado et al. (2014) found that Spanish children even at 4 years were sensitive to the presence of a dissenter, resulting in reduced trust in the majority opinion. Given that these studies differed in the methodology used and the topic addressed, no conclusions can be drawn regarding preschoolers’ receptivity to the presence of dissenters. However, it may be presumed that although non-conformist or dissenting individuals exist in all societies, the social reaction to them can vary depending on the cultural background, among other factors.

As mentioned earlier, several cross-cultural studies with adults indicate differences in the rate of deference to consensus. In particular, the research conducted from the individualistic-collectivistic cultural approach (Triandis, 1995, but see Xiao, 1999, for a critical view) yield findings showing that people from the so-called collectivistic cultures, such as East Asians, are usually more inclined to endorse the opinion of a group in consensus than people from so-called individualistic cultures, such as the U.S. (Bond and Smith, 1996). Cross-cultural studies with children are far fewer. To date, most research has been conducted in Anglo-Saxon countries (specially the U.S. and U.K.) with participants of European background. The work by Chen et al. (2013) is one of the few to compare children from two culturally different countries (U.S. and Taiwan) in tasks of labeling new objects within the majority-dissenter paradigm. The authors found that both the Taiwanese and the U.S. children were more likely to endorse the labels provided by the majority than by the dissenter, with no differences between countries. Other studies in the U.S. comparing American preschoolers of different cultural roots have found a stronger deference toward majority among Asian Americans than among Caucasian Americans, even when the majority made a clear incorrect choice (Corriveau et al., 2013). On the other hand, Chan (2011), Chan and Tardif (2013) compared children from the U.S. and Hong Kong in tasks of categorizing ambiguous and non-ambiguous objects and endorsing unexpected labels proposed by a teacher. Although her study was not concerned with consensus but with the influence of an epistemic authority, her results are relevant to the culture discussion. In particular, contrary to the conventional assumptions about the East–West cultures, Chan found that the Hong Kong preschoolers selectively relied on their prior beliefs to endorse or reject the information provided by the teacher, while the U.S. preschoolers were more compliant with the teacher’s claims. An additional example of the mixed findings with children from different cultures comes from a series of studies conducted with Spanish preschoolers facing labeling tasks (Guerrero et al., in press) or counting tasks (Enesco et al., in press). It should be mentioned that the Spanish culture, in terms of the cultural approach, is commonly considered as more collectivistic than American culture but still more self-oriented than East Asian cultures (Oyserman et al., 2002). According to this view, one would expect Spaniards to be in between these two cultures (U.S. and East Asian), in terms of rate of deference to consensus. However, contrary to this expectation, Guerrero et al. (in press) found that Spanish preschoolers did not show any bias toward majority when endorsing new labels; instead, they were as likely to support the majority claim as the dissenter claim. Also, in the study by Enesco et al. (in press), the Spanish preschoolers did not defer to the consensus but relied on their beliefs about counting procedures to accept or reject the majority’s assertions. In sum, the still few studies with young Spaniards reveal a fairly limited influence of consensus in their decisions. However, in view of the scarce and heterogeneous research, it would be daring to draw conclusions about the relations between cultural background and sensitivity or deference to majority.

The main objective of the present work is to study Chinese and Spanish children’s endorsement of the majority testimony regarding social matters, and whether the presence of dissenters modulates their trust in majority. We created two social decision contexts depicting events that are familiar and significant for children. In one scenario -social interpretation context-, children were presented with ambiguous social events open to interpretation. In the other scenario -moral judgment context-, children were presented with non-ambiguous events regarding the exclusion of a peer. In all cases, the informants (majority and dissenters) were adults acting as teachers in a school setting. We decided to present teachers instead of simply adults because teachers are important epistemic figures for children whom they attribute not only expert knowledge but also benevolence and honesty (Olson and Bruner, 1996). The strength of the majority in children’s decisions was assessed by two means: On one hand, we compared between-subject children’s choices in two conditions, with and without dissenter; on the other hand, we presented within-subject different majority’s view across trials of each context (e.g., approving exclusion or condemning exclusion, see **Table 1** for details). In contrast to most previous research, children were not only asked to make a two-option choice but also to provide reasoned justifications for their choices (i.e., why they agreed with this particular opinion). The assessment of children’ justifications may enhance our understanding of the influence of the testimony of others. We included a warm-up consisting of learning new words to determine children’s overall confidence in majority within this paradigmatic context of naïveté.

**Table 1:** Schematic summary of the procedure.

Context	No. Trial	Majority view	Dissenter/Alternative view
**Object labeling warm-up^a^**	1	Li-La (Reso) is object A	Li-La (Reso) is object B
	2	Feibo (Teno) is object A	Feibo (Teno) is object B
**Social interpretation^b^**			
Majority pro intentional	3	A was *intentionally* pushed by B	A *accidentally* fell off
Majority pro accidental	4	A *accidentally* fell off	A was *intentionally* pushed by B
**Moral judgment^b^**			
Majority approve	5	Peer exclusion is *right*	Peer exclusion is *wrong*
Majority condemn	6	Peer exclusion is *wrong*	Peer exclusion is *right*


*Trials were presented in the same fixed order across participants as shown below. ^a^Labels used in each language (Spanish in parenthesis). All objects and pseudo-words were made-up for this study and had been pre-tested with a small sample of 4–6 year-olds to ensure that children were unable to guess what the objects were and what the words meant. ^b^Drawings used in the two social contexts were adapted from McGlothlin et al. (2005), and Guerrero et al., in press.*

We targeted children from 4 to 6 years for two reasons. First, 4 years is the youngest age at which children clearly show sensitivity to the information offered within the testimony experimental paradigm. Second, the vast majority of previous research has been conducted with children aged 4–6 years, which provides us with a solid basis for comparison.

Based on previous research, we expected children to rely on the consensus significantly more when uncertain about what happened (social interpretation context) than when they recognize the situation as an explicit moral transgression for which they have strong prior beliefs (moral judgment context). We also expected the presence of a dissenter to decrease children’s endorsement of the majority view. We do not make specific predictions regarding cultural background, given the heterogeneous findings of previous studies. However, Chinese and Spanish societies may differ in aspects relevant to the issues addressed in this work. In particular, respect for elders and their teaching is a social value traditionally ascribed to the Chinese culture but not to the Spanish culture (Chao and Tseng, 2002). This alleged difference in children’s orientation to elders might influence the way Chinese and Spanish children weigh the testimony of teachers, particularly when it runs counter their own beliefs.

## Materials and Methods

### Participants

A total of 92 Chinese children aged 4–6 years (*M* age = 60 months; range: 44–75, 46 females), and 106 Spanish children 4–6 years (*M* age = 61 months; range: 51–75, 54 females) participated in the study. The Chinese children were recruited from two schools in the cities of Beijing and Hunan (China), and the Spanish children from two schools in Madrid (Spain), all of them serving middle SES families from the majority ethnic group. Informed consent was obtained from the parents of all the children, and only children who wanted to participate took part in the study. Participants were individually interviewed in their own language by a female researcher: a native speaker of Mandarin (fourth author) interviewed the Chinese preschoolers; and a native speaker of Spanish (fifth author) interviewed the Spanish children. Of this sample, 150 children participated in the experimental group (Chinese: *N* = 68, *M* age = 61 months; range: 44–75, 34 females; Spaniards: *N* = 82, *M* age = 62 months; range: 51–75, 42 females), and 48 children participated in the baseline group –see procedure- (24 children from each country with equal numbers of boys and girls, Chinese: *M* age = 57 months; range: 53–65; Spaniards: *M* age = 58 months; range: 54–63). In the experimental situation, children were randomly assigned to one of the two conditions with equal number of boys and girls: Dissenter (Chinese: *M* age = 59 months; range: 44–77; Spaniards: *M* age = 62 months; range: 53–75), and non-dissenter (Chinese: *M* age = 61 months; range: 46–75; Spaniards: *M* age = 61 months; range: 51–75) (see procedure below). Two-sample *t-*tests between cultural groups (Chinese, Spanish) confirmed no age differences in the total sample, *t*(192) = 1.55, *p* = 0.122, or in the experimental group, *t*(148) = 1.23, *p* = 0.223.

### Materials

**FIGURE 1:**
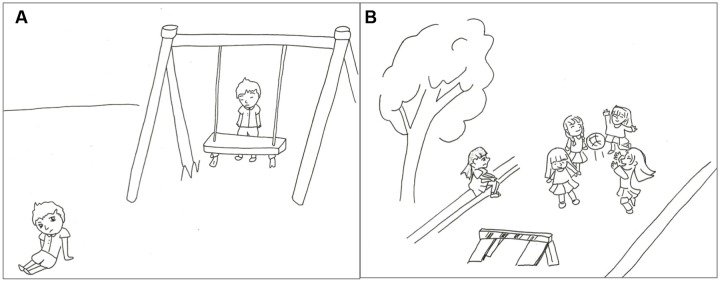
**Example of the drawings used for **(A)** the Social Interpretation and **(B)** the Moral Judgment tasks (adapted from McGlothlin et al., 2005, and Guerrero et al., in press).** The gender and ethnicity of characters depicted matched those of the participants.

Tasks were presented in a HP Pavilion 15″ portable. We used a series of digitalized drawings representing the different types of peer interactions (adapted from McGlothlin et al., 2005, and Guerrero et al., in press), see **Figure 1.** The gender and ethnicity of the characters depicted matched those of the participants. We presented short films (∼6 s) that featured the informants stating their opinion about the abovementioned situations. Both drawings and videos were different for each trial of each context.

### Procedure

Participants assigned to the *experimental group* began with the warm-up (two trials), and were then presented with the two social contexts in the following fixed order: Social interpretation context (two trials), moral judgment context (two trials) (see **Table 1**). All trials were structured as followed: first, the experimenter presented the situation to be evaluated; second, she offered the children further information from different groups of teachers (see Condition below), by showing them a video in which either three or four female adults (i.e., the teachers), depending on condition, made judgments about the situation; third, once the video had ended, the screen showed a still image of the teachers and the experimenter invited the children to evaluate the teachers’ claims, and whether or not they agreed with them; and finally, after their decision, the children were asked to give a justification.

#### Condition: Dissenter and Non-dissenter

Children from the experimental group were presented with one of the two conditions: The dissenter and non-dissenter conditions. In the dissenter condition (3 vs. 1), we presented three informants forming a numerical consensus and a dissenter disagreeing with the majority. The location of the dissenter on either the left or right of the numerical majority was alternated across trials. Also, the order in which informants spoke and were mentioned by the experimenter, either dissenter or majority first, was alternated across trials. This was done to avoid any memory effect on children’s responses. In the non-dissenter condition (3 vs. 0), we presented three unanimous informants. The amount of informants agreeing was kept constant across conditions (always three) to avoid potential confounding derivate from a varying number of majorities; that is, the condition varied only in the presence or not of a dissenter, but not in the number of the members of the majority. In both conditions, informants were female adults from the same racial group as the participants (either Chinese or Spanish) and were introduced as teachers; they all had similar body types and neutral facial expressions. It is important to highlight that the identity of informants systematically varied throughout the study so that children *always* saw different groups of persons in each trial and for each context. This was done to prevent participants from categorizing informants according to their previous opinion (either dissenter or majority view).

#### Contexts of Decision

##### Warm-up (object labeling context)

Children started with two warm-up trials. In the first one, participants were presented with two novel objects placed on a table in front of them. The experimenter (hereafter, E) said one of the two objects was named li-la/reso (Chinese and Spanish pseudo-words, respectively), but did not know which one; therefore, she had asked some teachers. She then presented a video that varied according to the condition. In the dissenter condition, participants viewed four females and the same two objects on a table while a voiceover asked: “Which of these things is a li-la?” Then, three of the females pointed at object A, while the other female pointed at object B. To ensure the children understood the events, E repeated the information: “These teachers said this is a li-la [E pointed at the three teachers in consensus and object A], and this teacher said that this is a li-la [E pointed at the dissenter and object B].” The non-dissenter condition was similar to the dissenter condition, except that the video featured only three females pointing at the same object. After watching the video, E asked the participants to choose either the majority view or the dissenter/alternative view. The same procedure was followed for the second trial, using two different novel objects and a different pseudo-word (fei-bo and teno for Chinese and Spanish children, respectively).

##### Social interpretation context

After the warm-up trials, the participants were presented the social interpretation and the moral contexts. In the social interpretation context, they completed two trials in which a purposely ambiguous social situation was presented (see **Table 1**). In the first trial (Majority pro intentional interpretation), the numerical majority interpreted what had happened as an act of harm. E presented a drawing showing two characters in a playground; one of them (e.g., María) is on the ground in front of a swing and the other (e.g., Julia) standing behind the swing (see **Figure 1**). E told the participant that she did not know what had happened, María had either fallen off the swing or Julia had pushed her off (for the sake of simplicity, hereafter the characters will be mentioned as A and B). E suggested seeing what the teachers said about this, and presented a video that varied across conditions. In the dissenter condition, three females state one by one that A was *intentionally* pushed off by B, and a fourth female states that A *accidentally* fell off the swing. In the non-dissenter condition, no dissenter was presented and a unanimous majority by turn provides the intentional interpretation. After watching the video, E repeated the information provided by the informants in the same order in which they spoke in the video (in the non-dissenter condition, she also stated the alternative view): “These teachers [pointing at the majority] said that Julia pushed María off the swing, and this teacher [pointing at the dissenter] said that María fell off.” Then, E asked: “Who do you think is right? The three teachers who say Julia pushed María off or the teacher who says that María fell off” (dissenter condition); “Do you think the teachers that say Julia pushed María off are right or wrong?” (non-dissenter condition). In the second trial (Majority pro accidental interpretation), the numerical majority interpreted the event as an accident. The situation depicted in the drawing consisted of two children in a playground, one of them laying on the ground and the other standing nearby. Note that the procedure for the second trial matched the one for the first trial except that the majority defended an accidental interpretation of the social interaction (Majority pro accidental interpretation). In the non-dissenter condition, all three females stated that A fell off accidentally, and in the dissenter condition, three females gave the accident version while a fourth female interpreted what had happened as intentional (A was pushed by B). The order of trial presentation (majority pro intentional interpretation first followed by majority pro accidental interpretation) was kept constant across participants.

##### Moral judgment context

Finally, we presented participants with two situations in which a child was explicitly excluded from a peer interaction (see **Table 1**). In the first trial, the majority approved the exclusion of the peer (Majority approve exclusion). The participants viewed a drawing showing three children at a birthday party, and another child sitting outside the house looking inside. E explained that one child (e.g., Lan-lan) had invited some children to her birthday party but that she hadn’t invited Hong-hong, the child sitting outside. Then, E invited the participant to see what some teachers said about what Lan-lan had done. In the dissenter condition, the participants viewed a video featuring four females, each of them voicing their opinion one by one, as in the social interaction context. Three females said what Lan-lan had done was right; the fourth female said it was wrong. In the non-dissenter condition, the story and procedure were the same except that the video showed only three females giving a unanimous opinion approving the exclusion of the peer. After watching both videos, E repeated the information provided by the informants, and then asked: “Who do you think is right? The three teachers who say that it’s OK not to invite her or the teacher who says it’s not OK” (dissenter condition); “Do you think the teachers that say it’s OK not to invite her are right or wrong?” (non-dissenter condition). In the second trial (Majority condemn exclusion), the majority condemned the peer exclusion. The participants were presented with a drawing showing three children playing on a playground on the monkey bars (see **Figure 1**). A child was sitting to one side, looking at the group but not playing. E explained that the group of children hadn’t let the other child play with them. Then, E invited the participant to see what some teachers had said about what the children had done. In the dissenter condition, three females condemned the exclusion while the fourth one approved it. In the non-dissenter condition the video featured only three females condemning the exclusion. The subsequent questions were analogous to those raised in the first trial. The order of trial presentation (majority approve exclusion first, followed by majority condemn exclusion) was the same for all participants.

The two social contexts just described were supposed to differ in their degree of ambiguity: The social interpretation context being open to interpretation (either as an accident or an action of harm) and the moral judgment context being unambiguously interpreted as morally wrong. To confirm these expectations, we tested an additional sample of participants from each country (24 Chinese, 24 Spanish) –*baseline group*, using the same materials as for the experimental group except that no informants were present. Children were presented first with the two trials of the social interpretation context, and then with the two trials of the moral judgment context. In each trial of both contexts, participants were shown the same drawings described before, and were then asked to evaluate the situations depicted and to justify their choices. The questions asked were: “What happened?” in the social interpretation context and “Was it right/wrong?” in the moral judgment context.

### Coding and Data Analyses

Our main dependent variable was majority endorsement, that is, whether children chose the majority option (scored 1) or the alternative option (scored 0). We also analyzed the children’s justifications. Here we measured (1) whether or not children provided a justification for their choices, and (2) the content of the justification. Regarding the former, “Don’t know” responses and circular arguments (e.g., “Because she fell down”; “Because it isn’t right”) were coded as non-justifications. In all contexts we found justifications based on *consensus* (explicit mention of the informants, e.g., “because all the teachers said so”), but other justifications were also provided, based on different arguments, specific to each context. In the social interpretation context, children’s explanations fell into two additional categories, which consisted of adding further information to the story in accordance with the participant’s interpretation: either as an intentional act of harm (*act-of-harm enriching*, e.g., “She pushed the other girl because she wanted to get on the swing”) or as an accident, non-intentional outcome (*accident enriching*, e.g., “The kid fell down because he was running”). In the moral judgment context, children’s justifications fell into two additional categories: *moral concerns* (references to fairness or empathy toward the excluded child, e.g., “I’m sure she feels sad because she would like to be there…”; “It’s not fair! They shouldn’t leave him alone”), and *reason for excluding* (providing a motive or rationale for the exclusion, e.g., “They didn’t let her play because she was too little and could be hurt”). In the warm-up object labeling context, children justified their choices referring to *consensus* and to *physical traits* of the novel object (relating the name to some trait of the object, e.g., shape, color).

We first present the general trends of children’s overall endorsement of the majority view and the number of children’s justifications in the experimental group. For this purpose, we analyzed children’s performance across all the contexts, including the warm-up trials. As mentioned before, the warm-up trials allowed us to evaluate children’s endorsement of the majority in a paradigmatic context of naïveté. Secondly, we focus on the main analyses conducted within each context, given the differential nature of the epistemic contexts. Here we explored children’s choices and the content of their justifications in accordance with the categories presented above. We used categorical analyses: one-sample binomial tests to assess (1) whether children from the experimental group endorsed the majority view more frequently than the alternative option and (2) whether children from the baseline group chose one of the two evaluations more often than chance; Chi-Square tests and Mann–Whitney tests to compare children’s choices and justifications between conditions and countries; McNemar tests for comparisons between trials.

## Results

Preliminary analyses indicated that girls and boys did not differ in their trust in majority within each context, Object labeling: χ^2^(1,300) = 2.67, *p* = 0.102, Social interpretation: χ^2^(1,300) = 0.07, *p* = 0.1796 and Moral judgment: χ^2^(1,300) = 2.59, *p* = 0.108. Likewise, we found no significant effects of the age of participants (in months) on their likelihood of siding with the majority (χ^2^ = 1.194, df = 1, *p* = 0.275), or order of presentation of the majority vs. dissenter view on children’s decisions to side with the majority, χ^2^(1,900) = 2.09, *p* = 0.148. Therefore, none of these variables were included in the analyses reported below. All reported *p-*values are two-tailed.

Considering the data collapsed across all six trials, children were significantly more willing to endorse the majority view than to choose the alternative, χ^2^(1,900) = 256.00, *p* < 0.001. Although this choice pattern remained constant across conditions [Non-dissenter: χ^2^(1,450) = 200.00, *p* < 0.001; Dissenter: χ^2^(1,450) = 72.00, *p* < 0.001], the presence of a dissenter resulted in a significant decrease in the majority choices, χ^2^(1,900) = 21.62, *p* < 0.001. No differences were found between countries in their general endorsement of majority, χ^2^(1,900) = 2.36, *p* = 0.125. However, analyses by condition revealed that the Chinese children were more likely to follow a unanimous majority (non-dissenter condition) than the Spanish children [χ^2^(1,450) = 5.83, *p* = 0.016], but they did not differ in the dissenter condition, χ^2^(1,450) = 0.00, *p* = 1.00.

Regarding justifications across all six trials, the presence of a dissenter significantly increased children’s general likelihood of providing a justification, χ^2^(1,900) = 4.55, *p* = 0.033. However, the Spanish children were more willing than the Chinese to give a justification for their choices (60 and 36% of occasions, respectively), χ^2^(1,900) = 52.94, *p* < 0.001.

Analyses on the general differences among contexts (with data collapsed across trials and conditions within each task) indicated that children’s endorsement of majority also depended on the context, Cochran’s *Q* test, χ^2^(2) = 57.13, *p* < 0.001. Pair-wise comparisons using continuity-corrected McNemar tests with Bonferroni correction showed that children were significantly less likely to side with majority in the moral judgment context than the social interpretation context and the object labeling warm-up (*ps* < 0.001), but that the majority choice was equally frequent in these two latter contexts (*p* > 0.0125). Note, however, that within each context, trials differed in what the majority opined, and this might have affected the children’s choices. Therefore, data within context must be analyzed before drawing any conclusion.

### Warm-Up (Object Labeling Context)

Children’s choices of the majority were unaffected by trial number, therefore we present analyses on data collapsed across this factor, McNemar tests, Spanish: *n* = 82, *p* = 1.00; Chinese: *n* = 68, *p* = 0.752. The Chinese and Spanish children did not differ in their overall confidence in majority in the labeling context, χ^2^(1,150) = 1.51, *p* = 0.470. Most of the participants’ responses sided with the majority view (non-dissenter condition: 93%, dissenter condition: 83%), although the presence of a dissenter significantly decreased their reliance on majority, Mann–Whitney test, *U* = 2.28, *n* = 150, *p* = 0.005.

#### Justifications

Regarding the justifications, the Spanish children (65% of occasions) were more willing to justify their choices than the Chinese (47% of occasions), χ^2^(1,300) = 9.35, *p* = 0.002. However, when they provided one, Chinese and Spanish children did not differ in the type of justifications (physical traits and consensus), χ^2^(1,170) = 0.001, *p* = 0.973. Specifically, children from both countries gave the same amount of physical trait justifications (42%) as consensus justifications (58%), regardless of the condition, dissenter vs. non-dissenter, in which they participated, χ^2^(1,170) = 1.23, *p* = 0.267.

### Social Interpretation Context

The participants in the *baseline group*, in which no informants were presented, showed no preferred interpretation in either trial (Binomial tests, Trial 1: *p* = 0.194; Trial 2: p = 0.470, ns = 48): Nearly half (42%) of the responses consisted of an accidental interpretation (A fell off by accident) and the remaining (58%) consisted of an intentional interpretation (A was pushed by B). There were no differences between nationalities, χ^2^(1,48) = 2.43, *p* = 0.297.

#### Majority Pro Intentional Interpretation

**FIGURE 2:**
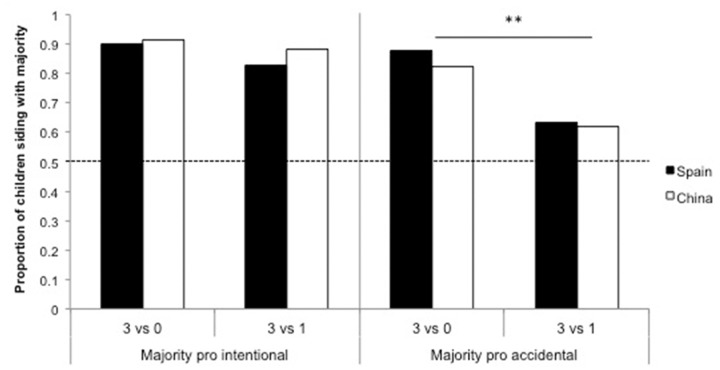
**Siding with the majority view in the social interpretation context.** Proportion of Chinese and Spanish children who sided with the majority in the two trials of the social interpretation context (majority pro intentional and majority pro accidental interpretation) by Condition, non-dissenter (3 vs. 0) and dissenter (3 vs. 1). ^∗∗^*p* < 0.01.

When the majority interpreted the situation as an intentional act of harm (A was pushed by B: Majority pro intentional interpretation) Chinese and Spanish children did not differ in their choices [χ^2^(1,150) = 0.343, *p* = 0.558], with most participants (88%) consistently siding with the majority. Moreover, children’s trust in majority was unaffected by the presence of a dissenter, who said that the character had fallen off by accident, χ^2^(1,150) = 1.01, *p* = 0.315 (see **Figure 2**).

#### Majority Pro Accidental Interpretation

When the majority provided an accidental interpretation (A fell off by accident: Majority pro accidental interpretation), again we found no differences between the Chinese and Spanish children, χ^2^(1,150) = 0.244, *p* = 0.622. Although the majority view was the most frequent choice in both conditions (binomial tests, non-dissenter: *p* < 0.001; dissenter: *p* = 0.038, *ns* = 75), when presented with the dissenter interpreting the situation as an act of harm, a significant third of participants supported this interpretation, χ^2^(1,150) = 10.01, *p* = 0.002 (see **Figure 2**).

#### Justifications

As found in the previous context, the Spanish children gave significantly more justifications than the Chinese in both social interpretation trials, majority pro intentional interpretation: χ^2^(1,150) = 24.53, *p* < 0.001; majority pro accidental interpretation: χ^2^(1,150) = 38.04, *p* < 0.001. While the Chinese children justified only 21% of their elections, the Spanish did so on 66% of the occasions. Given the small number of Chinese children who provided justifications, we were not able to run comparative analyses on the content categories. For this reason and because children from both countries did not differ in their choices of the majority, analyses were ran on data collapsed across country. Nevertheless, we also present the amount of justifications per country in parentheses. Overall, the proportion of consensus justifications (30%; Chinese: 7 out of 28; Spaniards: 35 out of 108) and the sum of the two enriching categories (70%; Chinese: 21 out of 28, Spaniards: 73 out of 108) remained constant across trials [χ^2^(1,136) = 0.029, *p* = 0.865] and conditions [χ^2^(1,136) = 0.053, *p* = 0.819]. Regarding the enriching categories, children gave more “act-of-harm enriching” arguments when the majority provided an intentional interpretation of the situation (58%; Chinese: 11 out of 18; Spaniards: 32 out of 55), and more “accident enriching” arguments when the majority gave an accidental interpretation (48%; Chinese: 6 out of 10; Spaniards: 23 out of 53). More interestingly, in this latter trial, we found a significant difference between conditions, as found in children’s pattern of choices: Children gave significantly more “act-of-harm enriching” justifications in the dissenter than in the non-dissenter condition, χ^2^(1,42) = 3.96, *p* = 0.04.

### Moral Judgment Context

In the baseline group, virtually all participants from both countries (Chinese: 96% and Spanish: 98%) condemned the peer exclusion in the two moral judgment trials in which they were asked to evaluate the exclusion events without informants.

#### Majority Approve Exclusion

**FIGURE 3:**
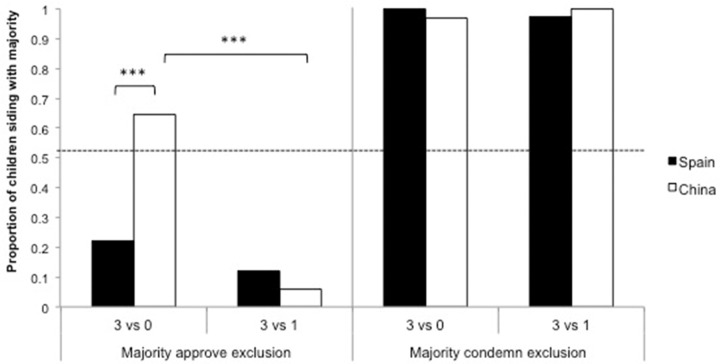
**Siding with the majority view in the Moral context.** Proportion of Spanish and Chinese children who sided with the majority view in the two trials of the moral judgment context (majority approve exclusion and majority condemn exclusion) by Condition, non-dissenter (3 vs. 0) and dissenter (3 vs. 1). ^∗∗∗^*p* < 0.001.

In the majority approve exclusion trial, the vast majority of both Chinese (94%) and Spanish (88%) children rejected the majority’s opinion when a ‘righteous’ dissenter was present, with no significant differences between countries, χ^2^(1,75) = 0.875, *p* = 0.349. However, an interesting difference emerged when no dissenter was present: Only 22% of Spanish children sided with the unanimous majority compared with 65% of Chinese children, χ^2^(1,75) = 14.01, *p* < 0.001 (see **Figure 3**). In other words, the Spanish children condemned the exclusion of a peer regardless of the condition in which they participated [χ^2^(1,82) = 1.378, *p* = 0.240]; Chinese children, by contrast, were notably more willing to condemn it in the dissenter than in the non-dissenter condition, χ^2^(1,68) = 25.76, *p* < 0.001.

#### Majority Condemn Exclusion

Not surprisingly, when the majority concurred with children’s previous beliefs, virtually all participants sided with the majority in the dissenter condition (100% of Spanish and 98% of Chinese) and the non-dissenter condition (98% of Spanish and 100% of Chinese), see **Figure 3.**

#### Justifications

Although the Spanish children (50%) tended to give a justification more often than the Chinese (39%) [χ^2^(1,300) = 3.137, *p* = 0.08], the distribution across the content categories (moral, reason for excluding, consensus) was similar for both countries, χ^2^(2,137) = 0.05, *p* = 0.975. Overall, children were significantly more likely to provide a moral justification for their choices than any other reason, regardless of the majority opinion on peer exclusion, either pro (76%) or against (83%) (see **Table 2**). However, we found interesting differences between conditions. Almost twice as many children provided a moral argument in the dissenter than in the non-dissenter condition. Conversely, in the latter condition, when presented with a unanimous majority, children were more reluctant, or less able, to give any kind of justification.

**Table 2:** Justifications in the moral judgment context.

		Justifications			
		
Condition	Trial	Moral	Reason for excluding	Consensus	Non-justification
Non-dissenter (3 vs. 0)	Majority approve	0.24	0.09	0.04	0.63
	Majority condemn	0.27	0.07	0.03	0.64
	Total	0.25	0.08	0.03	0.63
Dissenter (3 vs. 1)	Majority approve	0.44	0.04	0.04	0.48
	Majority condemn	0.51	0.01	0.05	0.43
	Total	0.47	0.03	0.05	0.45


*Proportion of justifications by Condition and trial. “Majority approve” refers to the trial in which the majority approved the exclusion of a peer. “Majority condemn” refers to the trial in which the majority condemned the exclusion of a peer. The row “total” refers to the mean proportion of justifications for each condition (collapsed across trial).*

## Discussion

This study examined Chinese and Spanish preschoolers’ use of consensus in two contexts of social judgment, one involving ambiguous events that were open to interpretation, the other context involving explicit (morally relevant) events of peer exclusion. In a between subject design, we assessed children’s trust in majority under two types of consensus: Unanimous (non-dissenter condition, 3 vs. 0) and non-unanimous (dissenter condition 3 vs. 1). As a warm-up, we included the paradigmatic context of new object labeling to assess children’s decisions in a state of naïveté, in contrast with the two social judgment contexts.

The general results indicated that children’s sensitivity to consensus varies depending both on the nature of the events (morally relevant or ambiguous) and the presence of a dissenter. On one hand, children were much more likely to endorse the majority view when they were naïve (object labeling warm-up) or uncertain (social interpretation context) about the situation, than when they already had strong beliefs (moral judgment context). On the other hand, participants were sensitive to the type of consensus, in that the presence of a dissenter reduced their willingness to side with the majority. Regarding the dissenter condition, the Chinese and Spanish children responded similarly: They consistently followed the lead of the majority, except when the majority approved the exclusion of a peer; in this case, most children sided with the dissenter. Nevertheless, in the non-dissenter condition, an unexpected picture emerged: Most Spanish children opposed the unanimous (pro-exclusion) majority while most Chinese sided with it. These results might suggest an interesting cultural difference in sensitivity to dissenters. Chinese children seemed to depend (at least, in part) on the presence of an “allied” dissenter to express their opinion; by contrast, Spanish children maintained their own criteria regardless of the type of consensus (with and without dissenter). It should be remembered that virtually all participants in the baseline group (without informants) from both countries condemned the peer exclusion, and not surprisingly, children from the experimental group also sided with the “righteous” majority rejecting exclusion, either unanimous or non-unanimous.

Overall, there is converging evidence that from early ages children selectively trust the testimony of others. Facing uncertainty, children are likely to use others’ testimony as evidence about reality; what some authors have described as a bias to copy when uncertain (e.g., Morgan et al., 2015). By contrast, children’s own criteria may prevail over the testimony of others when exposed to conflicting information (Chan, 2011; Einav, 2014) (see Mills, 2013 for a review). Our results with the Spanish children fit this general trend but this was not the case with our Chinese participants. One possible explanation could be that the Chinese “suspended” their own criteria in deference to a unanimous majority composed of teachers, and this might be due to a culture-specific pattern related to children’s attitude toward adults. As found in some cross-cultural research, Chinese children are not likely to challenge the authority of adults and show a respectful deference toward adults’ decisions even when they conflict with children’s own interests. In this line, in a study on moral reasoning in Chinese children, Fang et al. (2003) found that they elaborated arguments to justify an apparently arbitrary decision of the parents. An alternative interpretation is that Chinese children might be more inclined to infer that the adult informants approving the exclusion of a peer had a good reason to do so. In particular, these children might have attributed different meanings to the story, adopting a “moral” perspective when at least one of the teachers condemned the exclusion, or adopting a “social-organizational” perspective when nobody condemned the exclusion (for converging evidence, see Yau and Smetana, 2003). This interpretation is coherent with the findings by Killen et al. (2001) showing that preschoolers may reason about exclusion in terms of moral standards (e.g., when the exclusion is solely based on gender or race), or on the basis of conventional concerns (e.g., when the inclusion of the peer might interfere in the functioning of the group). Our data do not enable this question to be answered, in particular, whether deference toward adults makes children seek an alternative interpretation of the situation. A more in-depth interview might have helped to further explore these hypotheses.

It might also be asked why the Spanish children hardly took into account the opinion of a majority of teachers, irrefutable figures of epistemic authority. Previous studies have also shown Spanish children’s marked tendency to oppose a majority of adults making claims that run counter to children’s prior ideas (Cascado et al., 2014; Guerrero et al., in press). One of the studies comparing Spanish and US children within moral judgment contexts showed that the Spanish were more likely than their US peers to reject the majority claims in favor of their own criteria (Guerrero et al., 2013). More surprisingly, Spanish preschoolers rejected the opinion of not only a majority of adults but also their own teacher when suggesting non-conventional (but plausible) uses of common objects (e.g., using a bottle to drink soup or a fork to comb one’s hair; (Guerrero et al., in press). In the same line, within the context of number, Spanish children aged 4–7 opposed the claims of a majority of teachers regarding correct but unconventional procedures of counting (Enesco et al., in press). Regarding this apparent “lack of deference” to authority, we can speculate about the educational system and the subtleties of the child-teacher relationship in Spain, compared to other countries. One difference can be found in norms of discipline and conventions both inside and outside the classroom such as, for example, the rules about participation in class or the way they address teachers and other adults (e.g., Spanish children often completely ignore turn-taking). It is common that teachers describe their Spanish pupils as much more bustling, disrupting or unruly than pupils from other cultural roots (e.g., Asians, Latin Americans). The Talis survey (Spanish Ministry of Education, 2013) indicates that Spain is one of the OECD countries where secondary teachers waste more time dealing with students’ interruptions in the classroom. Although there are currently no similar surveys with preschool- and primary school teachers, it is conceivable that such classroom dynamic arise long before the secondary years.

It is tempting to frame these findings within theories of individualist-collectivist cultural values. From this perspective, East-Asian societies (e.g., Chinese, Japanese, and Korean) are usually described as emphasizing conformity, group connectedness and solidarity in order to preserve social harmony, whereas Western societies are characterized by emphasizing freedom and individual rights in order to assert self-worth in the face of collective pressure (Kim and Markus, 1999; see the meta-analysis by Bond and Smith, 1996). But, where should Spanish society is placed on this continuum? In principle, Spanish culture could be considered as essentially collectivistic (at least, following the traditional characterization of Latin cultures, see Triandis, 1989). However, our current results with Spanish children contradict this assumption. Indeed, this longstanding characterization of societies as collectivistic vs. individualistic entails a considerable oversimplification. To talk about supra-entities such as “East-Asian” culture has little meaning if this category includes people from countries as different as Japan, China or Korea, in terms of their present cultural values. As different authors have pointed (Killen and Wainryb, 2000; Turiel, 2004), this representation of cultures neglects the within-culture complexities of social interactions and the diversity of people’s social judgments. Killen et al. (2002) studied Japanese and US children’s judgments about the exclusion of a peer in different contexts, and found that context, rather than culture, was the most consistent predictor of children’s evaluations. Similarly, our findings reveal that the effect of culture is subordinated to the type of context and mediated by the presence of a dissenter.

Another interesting finding of the present study is related to the social interpretation context. In this context, when the majority of informants opted for the interpretation of an act of harm (i.e., A was pushed by B), children consistently sided with them, and they did so regardless of the presence of a dissenter (whose opinion was that A fell off by accident). Interestingly, the picture was slightly different in the reverse situation in which the majority provided a non-intentional interpretation. In this case, even though children continued to side with the majority in both conditions, the presence of a dissenter interpreting the situation as an act of harm resulted in a third of participants supporting this view. This bias was displayed by both Chinese and Spanish children even when, as a group, neither presented a preferred interpretation of what happened in the absence of informants, as it shown in the baseline group. This finding is not discussed in much depth here because it was not part of our research question. However, it is worth noting that this could be interpreted as evidence of a negativity bias. There is some empirical evidence that children, in the same way as adults, are more likely to attend to negative than positive information in a variety of contexts (for a review see Vaish et al., 2008, but see Boseovski and Lee, 2008 for different findings). Some studies have revealed that from preschool age, children talk and recall negative events in a more sophisticated way than positive events (Lagattuta and Wellman, 2002; Fivush et al., 2003). This asymmetry might also be present in the context of the testimony of others, in that children may be influenced by the valence of the information. Alternatively, this finding could be due to the fact that the interpretation as an act of harm is informatively richer than the accident interpretation because the former implies a two-party interaction driven by a psychological motive, and the latter depicts a non-social accidental event. In any case, it would be interesting to further investigate the valence of information provided by others within the field of testimony, particularly, whether positive information (e.g., B is helping A to rise up) biases in the same way as negative information.

We will now discuss the children’s justifications. Overall, these results give additional support to the idea that children selectively attend to consensus, and that their base knowledge is a key variable to decide whether or not to endorse the testimony of others. In coherence with children’s general endorsement of the majority view across contexts, a consensus-based justification was provided (e.g., “because all the teachers said that”) on about 60% of the occasions in the labeling context (no prior knowledge), 30% in the social interpretation context (uncertainty), and less than 10% in the moral judgment context (strong convictions). The analyses of the justifications also provided evidence about children’s sensitivity to dissenting opinions. In the dissenter condition, that is, when two opposite views were represented, children were more willing, or able, to provide a justification. This was particularly evident in the moral judgment context, where both Chinese and Spanish children gave significantly more moral arguments in the dissenter condition than when only one option was available (non-dissenter condition), and this occurred regardless of whether the informants condemned or approved exclusion. This finding suggests that facing conflicting opinions may encourage children to reason about or seek an explanation for dissenting beliefs. The only difference between countries was found in the amount of justifications, with Spanish children providing, overall, more arguments than Chinese children. This may be interpreted in line with the hypothesis of a cultural difference in children’s orientation toward adult authority. It seems plausible that children from both countries saw the experimenter (and not only the informants in the video) as an authority, but that the Chinese were more reluctant than the Spanish to express themselves freely in front of an adult.

The current study has some limitations and raises interesting questions for future research. First, in this kind of paradigm, the informants (individuals transferring the knowledge) do not provide the social cues that are essential in the process of social communication. This might preclude children from understanding what is going on. For instance, hearing a group of teachers condoning the exclusion of a peer with no further justification is probably perplexing for children, and we cannot rule out that our participants would have responded differently if given some explanation or rationale (e.g., they were not friends and Li-li invited only her very close friends). A few studies provide a good illustration of the subtle differences that arise when children are given –or not-information about the circumstances or reasons underlying the informants’ behaviors and how this relates with the type of knowledge implied (Nurmsoo and Robinson, 2009a,b). A more general concern about this paradigm has been well noted by Howe (2014), who stated that this type of experimental setting is concerned with children’s acceptance or rejection of verbal information from others, rather than social influence on children’s learning and knowledge acquisition. Following Hodges’ (2014) suggestions, to better comprehend the way children acquire or revisit their prior ideas in light of the testimony of others, future research in this field should create more realistic settings in which children are allowed to exchange ideas with others, that is, a setting preserving the conversational nature of an interaction among real people.

Second, like most research in this field, our study focuses on a limited age range and, in this sense, lacks a genuine developmental perspective. We can assume that the socio-cognitive changes that take place during childhood do affect the way children weigh different variables related to the informants (e.g., accuracy, epistemic authority), the context (e.g., serious, pretend), the content of information, and the degree of congruity with one’s ideas. Also, as children develop and individual differences strengthen, their sensitivity to consensus might take different trajectories, more or less deferential or oriented to conform with consensus, more or less tolerant of dissent/deviance, as the literature with adults has shown (for a review see Hodges, 2014). Since no previous studies have adopted a comprehensive developmental view on these aspects, this deserves extensive research.

As a whole, our data suggest that from early ages, children take into account a series of variables when deciding whether or not to follow the lead of the majority. One of the relevant factors is the presence of a dissenter, which seems to stimulate children’s willingness to evaluate the information. In the face of conflicting claims, our participants not only reduced their confidence in majority but were also more likely to provide a reasoned justification for their choices. Children’s decisions to use the testimony of others were partly conditional upon their degree of certainty about what happened: When having a clear interpretation of the situation, they were far less influenced by consensus than when facing an ambiguous event. Interestingly, this general trend seemed to be modulated by culture, in that Chinese children were less willing than the Spanish to stick to their guns vis-à-vis a unanimous majority. However, this picture is highly provisional given the scarcity of cross-cultural studies on the testimony of others, and in particular, within the social domain. Crucially, empirical efforts should be directed at unraveling the developmental, individual and cultural factors affecting the way children evaluate the information provided by others.

## Author Contributions

IE, CS-E, and SG contributed equally to this work. SQ and SG authors also contributed equally to this work.

## Conflict of Interest Statement

The authors declare that the research was conducted in the absence of any commercial or financial relationships that could be construed as a potential conflict of interest.
